# The dual burden of malnutrition and its associated factors among mother-child pairs at the household level in Ethiopia: An urgent public health issue demanding sector-wide collaboration

**DOI:** 10.1371/journal.pone.0307175

**Published:** 2024-11-04

**Authors:** Muluken Yigezu, Abdu Oumer, Bereket Damtew, Dereje Birhanu, Sewnet Getaye Workie, Aragaw Hamza, Anteneh Atle, Natnael Kebede

**Affiliations:** 1 Department of Public Health, College of Medicine & Health sciences, Dire Dawa University, Dire Dawa, Ethiopia; 2 Department of Nutrition & dietetics, School of Public Health, College of Medicine and Health Sciences, Bahir Dar University, Bahir Dar, Ethiopia; 3 Department of Epidemiology and Biostatistics, school of public health, College of Medicine & Health Sciences, Debre Berhan University, Debre Berhan, Ethiopia; 4 Department of Anesthesia, College of Medicine & Health sciences, Dire Dawa University, Dire Dawa, Ethiopia; 5 Department of Public Health, College of Medicine & Health Sciences, Wollo University, Dessie, Ethiopia; Imam Abdulrahman Bin Faisal University, SAUDI ARABIA

## Abstract

**Introduction:**

The coexistence of under-nutrition and over-nutrition in developing countries like Ethiopia results in the "mother-child pair double burden of malnutrition," with children experiencing either stunting, wasting or underweight while mothers face overweight or obesity. This poses a major public health challenge, prompting global health organizations to prioritize the issue and urge governments to act quickly. Despite this, there is a lack of research in Ethiopia on the double burden of malnutrition among mother-child pairs at the household level and the factors that worsen it.

**Objective:**

To assess the magnitude of double burden of malnutrition and its associated factors among mother-child pair at household level in East Ethiopia, 2022.

**Method:**

A community-based cross-sectional study was conducted in East Ethiopia from April 15 to June 11, 2022. Multi-stage sampling was used, and data were collected through structured interviews. Child nutrition indicators were processed using WHO Anthro software. Binary logistic regression analysis was performed, calculating both crude and adjusted odds ratios to assess associations. Variables with a P value <0.05 in multivariable analysis were deemed statistically significant.

**Result:**

The study revealed that coexisting malnutrition rates for (OM/SC), (OM/WC), and (OM/UC) were 8.5%, 7.0%, and 7.9% respectively. The double burden of malnutrition among mother-child pairs was found to be 12.3% [95% CI: 10.7, 13.7]. Marital status (divorced) [AOR = 1.80; 95% CI: 1.15, 2.82], child birth order (fourth or above) [AOD = 1.88; 95% CI:1.08, 3.26], number of under-five children in the household (five or more under-five children) [AOR = 1.58; 95% CI: 1.04, 2.39], poor maternal and child dietary diversity score [AOR = 2.76; 95% CI: 1.71, 4.45] and [AOR = 8.66; 95% CI: 4.85, 15.44], respectively, household food security status (food insecurity) [AOR = 3.68; 95% CI: 2.36, 5.75], and maternal stature (short stature) [AOR = 2.39; 95% CI: 1.65,3.45] were factors significantly associated with this burden.

**Conclusion:**

The study emphasized the double burden of malnutrition affecting both mothers and children, a major public health concern in the area. Early-life nutrition is vital in preventing childhood under nutrition and adult obesity, leading to this dual burden. Breaking the cycle of malnutrition across generations is crucial. Policy makers should prioritize improving child nutrition and maternal health, stressing early-life nutrition to address the mother-child double burden of malnutrition.

## Introduction

The double burden of malnutrition occurs when under nutrition and over nutrition coexist, impacting individuals, households, or communities [1]. At the individual level, this manifests as a combination of stunted growth and overweight or obesity in children [1]. Household-level double burden involves the presence of undernourishment in one member and over nutrition in another within the same household. Community-level double burden is characterized by both ends of the malnutrition spectrum coexisting in the same community, particularly among children [2].

The dual burden of malnutrition is a significant challenge faced by many nations, marked by the coexistence of under nutrition and overweight or obesity, along with non-communicable diseases [3]. This phenomenon is prevalent in low- and middle-income countries, where households may have overweight or obese mothers and undernourished children, underscoring the complexity and importance of addressing this issue [4].

Global statistics from the Food and Agriculture Organization (FAO) reveal the alarming prevalence and impact of malnutrition worldwide. Millions of children under five suffer from stunted growth, while millions more succumb to overweight or obesity-related complications annually [5]. The global burden of overweight and obesity contributes to numerous deaths and disability-adjusted life-years, highlighting the urgent need for comprehensive interventions to combat this public health crisis [6, 7].

The global issue of the mother-child double burden of malnutrition affects nearly one in three individuals worldwide [8]. The management costs associated with addressing this issue amount to billions of dollars annually, emphasizing the urgent need for effective strategies to tackle the dual burden of malnutrition on a global scale [9–11].

Increasing rates of underweight and overweight in developing countries are a pressing concern driven by shifting dietary habits, disease burdens, and demographic transitions [12, 13]. As societies undergo economic progress and urbanization, there is a notable rise in overweight, obesity, and non-communicable diseases, particularly among older individuals. These shifts in population structures underscore the complex interplay between nutrition, health outcomes, and socio-economic development [14].

The global challenge of double burden malnutrition, encompassing both undernutrition and overweight, poses a significant obstacle in developing countries, straining already fragile health systems [15, 16]. WHO highlights the importance of addressing dual malnutrition burden to achieve UN nutrition and sustainable development goals, focusing on the African region from 2019–2025 WHO emphasizes the importance of addressing this dual malnutrition burden to achieve UN nutrition and sustainable development goals, with a specific focus on the African region from 2019–2025. Efforts to combat the dual burden of malnutrition are crucial for improving health outcomes and advancing global health equity [17–19].

In Sub-Saharan Africa, the prevalence of overweight or obesity affects 20–50% of the urban population [20], with a projected 75% of the global obese population residing in low-income countries by 2025 [21]. Ethiopia, as a country in Sub-Saharan Africa, grapples with a high burden of malnutrition despite efforts to reduce hunger and under nutrition. The ongoing challenge of malnutrition in Ethiopia underscores the need for sustained public health interventions to address this pressing issue [22].

The global issue of malnutrition is a critical concern for international health organizations, with the World Health Organization (WHO) prioritizing the Double Burden of Malnutrition (DBM) through "double-duty actions". In Ethiopia, the government has set ambitious targets to end child undernutrition by 2030, supported by the recent development of the National Food and Nutrition Policy [23]. Research in Ethiopia aims to bridge the gap in knowledge regarding the double burden of malnutrition among mother-child pairs, moving beyond previous studies focusing on maternal overweight/obesity and child stunting to provide a more comprehensive understanding.

Global nutrition targets for 2025 seek to reduce childhood stunting, wasting, low birth weight, and anemia in women of reproductive age, aligning with initiatives like the Maternal, Infant, and Young Child Nutrition (MIYCN) program and the Global Action Plan on Non-Communicable Diseases (NCDs). These efforts aim to combat obesity and other NCD risk factors by 2025 [24]. Addressing the complex issue of malnutrition requires a multi-faceted approach that considers the interconnected nature of nutrition challenges in Ethiopia and other low-income countries, emphasizing the importance of comprehensive strategies to improve health outcomes.

## Materials and methods

### Study area and period

The study was conducted in Eastern Ethiopia from April 15, 2022, to June 11, 2022. Eastern Ethiopia comprises five main administrative states. Three districts were randomly selected: Harari regional state, Dire Dawa city administration, and west Hararge zone.

In 2007, the population of Western Hararge in Eastern Ethiopia was 2,723,850, showing an increase of 48.79% from 1994. The region had 580,735 households and a population density of 151.87 individuals per square kilometer [25]. The Harari region, with 183,344 residents, included 19 urban kebeles and 17 rural kebeles, with a diverse ethnic population, the majority of whom lived in urban areas [26]. In the same region, Dire Dawa in Eastern Ethiopia had 9 urban and 38 rural kebeles, with a population of 341,834, comprising diverse ethnic groups [25].

### Study design and population

A community-based cross-sectional study was conducted among 1,668 mother-child pairs in the study area. The source populations for this study were all mothers who have a child (6–59 month) in East Ethiopia, with the study population including all selected households that have mothers with children (6–23 months) in the selected kebeles of East Ethiopia. Mothers who have a child from age 6–23 month live at least for six months in selected kebeles in Eastern Ethiopia during the study period were included in this study. However, Pregnant women with a delivery within 2 months before the survey and those who were unable to respond during the data collection period were excluded.

### Sample size determination and sampling procedures

The sample size was determined by using a single population proportion formula by considering the following assumptions: The anticipated population proportion (P) was 23 which were for coexistence of overweight or obese mothers with stunted child in Ethiopia [8], 95% confidence (Z = 1:96), 3% margin error (d), design effect of 2, and non-response rate of 10% giving a final sample size of 1668. The sample size after adding 2 as a design effect was 1,513. After adding 10% non-response rate the final sample size was 1,668.

Multistage random sampling technique was used. From five administrative states in East Ethiopia, three districts (East Hararge zone, Harari regional state, and Dire Dawa administration) were selected using simple random sampling. Population size, number of households, and total number of kebeles (the smallest administrative unit) were obtained from each district’s city administration office. Kebele and household lists were organized by house number for the selected districts. Kebeles were then randomly selected from each administrative state after stratifying them as urban and rural. The number of kebeles selected from each stratum was determined by taking 20% of the total kebeles as a rule of thumb.

Finally, in each kebele a fixed number of households were drawn by systematic random sampling technique. Then after allocating the number of households in each kebele proportionally, the number of the households were identified from the central place particularly kebele administrative offices. The K value was determined by dividing the source population by the sample size, *K = N/n*. The first household was selected randomly by using the lottery method and the samples were taken every 3 units (households) for the data collection afterward. During the data collection process, households that did not meet the specified selection criteria were skipped and the next household on the list was chosen.

### Operational definition

The double burden of malnutrition: is defined by the co-existence of serious levels of under- and over nutrition [27].

Double burden of malnutrition in mother child pair is the coexistence of under nutrition (either stunting, underweight or wasting in children) and overweight/obesity in mothers in the same household [28].

Overweight: describe by body mass index (BMI) from 25–30 [12].

Obesity: BMI 30kg/m^2^ or more [12].

Stunting: Height for age value less than two standard deviations of WHO child growth standard media [29].

Wasting: Is defined as low weight-for-height value less than two standard deviations of WHO child growth standard media [30].

Household food security status: Household experiences none of the food insecurity (access) conditions, or just experiences worry, but rarely was considered as secured, otherwise considered food insecure [17].

### Data collection tools and procedures

Data were collected using pretested structured interview administered questionnaire to obtain general information about households, demographic and socioeconomic factors and anthropometric measurements. The questionnaire first translated from English to Amharic, “Afan Oromo”, “Somaligna”, “Haderigna” and then translated back to English by language experts to maintain consistency. Finally, the questionnaire was administered in Amharic, “Afan Oromo”, “Haderigna”, & “Somaligna” language for data collection.

Nutritional status of mother-child pair (both under-nutrition and over-nutrition) was assessed using anthropometric measurement. A calibrated and portable personal weight scale was measure weight. During measurement, any heavy clothes, shoes, socks, and items from their pockets was removed from participants. Before measurement, the scale be zeroed, and the study participant’s stand in the center without any support until the result is recorded. Zero adjustment on the scale also made before taking the next measurement [31]. Height was measured using a calibrated stadiometer with headboard. During height measurement, the data collector wiped to clean the stadiometer and explained to the study participant about the measurement.

Participants were asked to take off their shoes, wear light clothes, and stand with their back to the stadiometer and look forward directly with their arms hanging loosely at their sides. The back of their feet, calves, buttocks, shoulders, and the back of their head made to be in contact with the stadiometer. Finally, the participants was asked to take a deep breath and hold [32]. All measurements were taken to the nearest 0.1 kg and 0.1 cm for weight and height measurements, respectively. If the first two measurements of weight or height were different by ≥ 0.5 kg or ≥ 0.5 cm respectively, another measurement was taken by a third person [33].

Recumbent length was measured lying down for children younger than 24 months and for those with unknown age and less than 87 cm. Child nutritional status was determined using the WHO 2006 reference standard to calculate standard deviation z-scores. Childhood undernutrition can be defined as height-for-age, weight-for-age and weight-for-height z-scores below −2 standard deviations from the WHO reference standard, being stunting, underweight and wasting, respectively. Childhood overweight will be defined as weight-for-height >+2 standard deviation z-score [34].

Food insecurity was measured by the household food insecurity access scale (HFIAS) which consists of nine occurrence question that represent a generally increasing level of severity of food insecurity (access), and nine ‘‘frequency–of-occurrence” question that will be asked as a follow-up to each occurrence to determine how often the condition occurred during the previous 4 weeks (last one month). The respondent first asked an occurrence question that was, whether the condition in the question, happened at all in the past four weeks (yes or no) if the respondent answers ‘‘yes” to an occurrence question, a frequency-of occurrence question was asked to determine whether the condition happened rarely (once or twice), sometimes (three to ten times) or often (more than ten times) in the past four weeks [34].

Household dietary diversity (HDDS): Each food group is assigned a score of 1 (if consumed) or 0 (if not consumed). The household score was range from 0 to 12 and is equal to the total number of food groups consumed by the household [35]:

HDDS=Sum(A+B+C+D+E+F+G+H+I+J+K+L)

The average household dietary diversity score for the population of study can be calculated as follows:

$$Average\;HDDS=\frac{Sum(HDDS)}{Total\;number\;of\;households\;surveyed}$$

Maternal dietary diversity assessment: assessed by an interviewer administered structured questionnaire which is adapted from different studies was used to collect data. Standard food and nutrition technical assistant (FANTA) tools were used for dietary diversity questionnaires based on 24-hour recall from subjects or caregivers. The questionnaires have a score of 10, which is classified as: low dietary diversity score (0–4), high or good dietary diversity score (5–10) [36].

Child minimum dietary diversity: The minimum dietary diversity (MDD) score for children 6–23 months old is a population-level indicator to assess diet diversity as part of infant and young child feeding (IYCF) practices. This indicator is part of the suite of complementary feeding indicators for IYCF developed by WHO and UNICEF to provide simple, valid, and reliable metrics for assessing IYCF practices at the population level. The eight food groups are: 1) Breast milk, 2) Grains, white/pale starchy roots, tubers and plantains,3) Beans, peas, lentils, nuts and seeds,4) Dairy products (milk, infant formula, yogurt, cheese), 5) Flesh foods ((meat, fish, poultry, organ meats), 6) Eggs,7) Vitamin-A rich fruits and vegetables & 8) Other fruits and vegetables [37].

Child minimum dietary diversity is measured as a percentage of children 6–23 months of age who consumed foods and beverages from at least five out of eight defined food groups during the previous day. The scoring system used to assess dietary diversity are categorized into two groups: low/poor dietary diversity score (≤4) and good/minimum dietary diversity score (≥5). The low/poor category is characterized by a lack of variety in the diet, while the good/minimum category represents a minimum level of dietary variety. In the context of the questionnaires, a score of 8 falls within the good/minimum category, indicating that the individual has achieved a minimum level of dietary variety. This suggests that the person consumes a diverse range of foods, providing them with a balanced and nutritious diet [37].

Behavioral factors assessment: Alcohol consumption, tobacco, and other substance use was assessed and categorized based on the WHO stepwise approach to chronic disease risk factor for surveillance. Physical activity, which includes, work-related and leisure/spare time physical activities; were measured by global physical activity questionnaire (GPAQ) [38].

### Data quality assurance

The data collection instrument was translated into local languages and back to English to ensure accurate understanding by participants. A two-day training session was provided to health officers and nutrition professionals on data collection procedures. Pre-testing was conducted with 5% of participants to verify the effectiveness of the tool, and on-site supervision was carried out daily to ensure completeness and consistency of questionnaires.

To further ensure data integrity, the data collector managed the setup for providing and retrieving questionnaires from study participants. Structured interviews were conducted in a confidential and private setting to minimize bias. Simple frequencies and cross-tabulation were used to address missing values and outliers, and the collected data were cross-referenced with hard copies to ensure accuracy and consistency. These measures were put in place to maintain the quality and reliability of the collected data throughout the process.

### Data processing and analysis

The data were coded, cleaned, edited and entered into Epi data version 3.1 to minimize logical errors and design skipping patterns. Then, the data were exported to STATA for analysis and finally data analyses was conducted with Stata/SE version 14.0. Descriptive statistics such as frequency, percentage, mean values, and standard deviations was computed for respondent characteristics and other measured study variables.

To evaluate the nutritional status of children, Z-scores for height-for-age, weight-for-age, and weight-for-height were calculated using WHO Anthro version 3.2.2.1 software. Additionally, the mother’s nutritional status was assessed by determining her body mass index (BMI). In the logistic regression model employed, the focus was on identifying the presence of the mother-child pair double burden of malnutrition (MCDBM), which was categorized as a binary variable. Instances where MCDBM was present were designated as "1", while those without this burden were assigned a value of "0". Therefore, households with mother-child pairs experiencing the Mother-Child Double Burden of Malnutrition (MCDBM) may involve a situation where the mother is overweight or obese (BMI >25–29.9 kg/m2 for overweight and ≥30 BMI for obese) while the child is undernourished with stunting/wasting/underweight (<-2SD Z-scores).

Bi-variable analysis was carried out with all factors to determine associations within the households. A multiple logistic regression was performed by using inter method to see factors considerably related to MCDM, as well as independent variables supported univariable logistic regression results (cut-off point p ≤0.25).

The final model was built based on inter method of multiple logistic regression (p ≤ 0.05). Variables with p > 0.25 were dropped from the multi-variable analysis. Hosmer- Model fit to the data (validity and reliability) was assessed by the likelihood ratio χ2 test and Hosmer-Lemeshow goodness-of-fit test. ROC curve also checked adjusted odds ratio with 95% CI was estimated to identify the factors associated with MCDB using multivariable logistic regression analysis.

Level of statistical significance was declared at p-value <0.05. Variables that have a p-value of < 0.25 after binary logistic regression were fitted into a multivariable logistic regression model to identify the independent contribution of each variable. If the P- value is <0.05 for a given variable after multivariable analysis, the variable is significant, it has an association with the outcome variable, and the degree of association between variables were measured using odds ratio.

### Variables of the study

The dependent variable was overweight /obese mother with under-nourished (either wasted or stunted) children live in the same household and independent variables Socio-demographic characteristics:-Age, Sex, Religion, ethnicity, educational status of mother, Educational status of Father, Occupation, family size; Maternal and Child characteristics:- stature of the mother, Breast-feeding practice, Birth order, child minimum dietary diversity, House hold dietary diversity; maternal dietary diversity; Physical activity, which includes, work-related and leisure/spare time physical activities; Behavioral factors:-Alcohol consumption, tobacco use; Food security status:—Household food insecurity.

### Ethical approval

In order to conduct this research, the authors tried to address the Declaration of Heliyons Ethical principles for medical research. First, ethical clearance was obtained from Dire Dawa University Institutional Review Board (IRB). A protocol number given by approving ethical committee is DDU-IRB-2022-067. A formal letter for permission and support was written to Dire Dawa city administration Education Bureau from Dire Dawa University and finally to selected high schools. Permission was also be secured from the respective high schools. All the study participants were informed about the aim of the study, their right to refuse. All participants were informed of the aim of the study and their right to refuse the study. Informed voluntary written and signed consent was obtained from all study participants prior to data collection. For illiterate and minor participants informed voluntary written and signed assent was obtained from their parents and/ or legal guardian. The respondents were also be told that the information obtained from them was treated with complete confidentiality and do not cause any harm on them. The participants were allowed to consider their participation and given the opportunity to withdraw from the study if they wished to do so.

## Results

### Baseline socio demographic characteristics

A total of 1668 participants took part in the study, yielding a response rate of 100%. The majority of the participants, constituting about three-fourths of the total, were 1219 (73.1%). In terms of marital status, the majority, 1219 (73.1) were married, while only 88 (5.3) were unmarried. When considering the age of the mothers, the largest group of study participants, 792 (47.5%), fell within the 30–34 age bracket. This was followed by 529 (31.7%) in the 25–29 age range, with only about 167 (10%) being aged 35 or older and 180 (10.8%) falling within the 15–24 age group. In relation to the educational status of the mothers, 474 (28.4%) had attained primary education, while 132 (7.9%) were unable to read and write.

As for the occupation of the mothers, the majority, 510 (30.6%), were engaged in merchant activities, while only 135 (8.1%) were employed in government organizations. The majority of respondents, comprising 576 (34.5%), had five or more family members in their household, while 557 (33.4%) had between one and two family members. Over half, 899 (53.9%), of the respondents had one to two under-five children. In terms of residency, more than half, 1012 (60.7%), lived in urban areas. When considering maternal height stature, 708 (42.4%) were classified as short. (See Table 1).

**Table 1:** Demographic characteristics for double burden of malnutrition and its associated factors among mother-child pair at household level in Dire Dawa city, Eastern Ethiopia, 2022 (n 1,668).

NO	Variables	Response category	Frequency (%)
01.	**Marital status**	Married	1219(73.1)
		Unmarried	88(5.3)
		Divorced	253(15.2)
		Windowed	108(6.5)
02.	**Mothers educational Status**	can’t read & write	132(7.9)
		can read & write	342(20.5)
		primary (grade 1-8^th^)	474(28.4)
		secondary (grade 9- 12^th^)	411(24.6)
		college & above	309(18.5)
03.	**Mothers’ occupation**	No job	144(8.6)
		Private employee	330(19.8)
		Government employee	135(8.1)
		Farmer	411(24.6)
		Merchant	510(30.6)
		Daily labor	138(8.3)
04.	**Family size**	1–2	557(33.4)
		3–4	535(32.1)
		≥5	576(34.5)
05.	**Number of under-five children in the household**	1–2	899(53.9)
		3–4	448(26.9)
		≥5	321(19.2)
06.	**Mother’s age in year**	15–24	180(10.8)
		25–29	529(31.7)
		30–34	792(47.5)
		≥35	167(10)
07.	**Residency of participants’**	Rural	656(39.3)
		urban	1012(60.7)
08.	**Maternal height stature**	Normal	960(57.6)
		short	708(42.4)

### Diet related characteristics

Only about one-third of mothers, specifically 33.3%, scored well on dietary diversity, leaving the majority with a poor (low) intake of 66.7%. Likewise, a similar pattern was observed in households, with only 32.3% achieving a good dietary diversity score. The data also revealed that a significant proportion of households, 67.5%, were classified as food insecure, indicating a concerning level of food insecurity among the surveyed population. (See Table 2).

**Table 2:** Diet related characteristics of the participants for double burden of malnutrition and its associated factors among mother-child pair at household level in Dire Dawa city, Eastern Ethiopia, 2022 (n 1,668).

No	Variables	Response category	Frequency (%)
01.	**Maternal dietary diversity (MDDS)**	good	555(33.3)
		poor	1113(66.7)
02.	**House-hold dietary diversity score (HDDS)**	good	539(32.3)
		poor	1129(67.7)
03.	**Household Food security status (HFS)**	Good	542(32.5)
		poor	1126(67.5)

### Lifestyle/behavior related characteristics

The data reveals high rates of smoking among the participants, with over 41.5% reporting past smoking habits, nearly 29% currently smoking, and 30.6% recently started smoking. Alcohol consumption was also prevalent, with approximately 27% having consumed alcohol in their lifetime and 15.6% within the past month. These findings highlight the need for public health interventions to address smoking and alcohol use.

Additionally, the data shows low engagement in vigorous-intensity work activities and moderate-intensity sports, indicating a lack of regular exercise. The low participation in active transportation methods like walking or cycling further suggests a sedentary lifestyle. Interventions promoting physical activity, especially vigorous-intensity work activities, moderate-intensity sports, and active transportation, could improve overall health and well-being in the community. (See Table 3).

**Table 3:** Lifestyle characteristics of the participants for double burden of malnutrition and its associated factors among mother-child pair at household level in Dire Dawa city, Eastern Ethiopia, 2022 (n 1,668).

S. N	Measurement items for behavioral characteristics	Yes (%)
1.	Have you ever smoked tobacco or used smokeless tobacco? (n = 1668)	693 (41.5%)
2.	Do you currently use (smoke, sniff or chew) any tobacco products such as cigarettes, cigars, pipes, chewing tobacco or snuff? (n = 1668)	480 (28.8%)
3.	For how long have you been smoking or using tobacco daily? (n = 600)	
	Less than 1 month	510 (30.6%)
	More than one month	90 (5.4%)
	**Alcohol consumption**	
4.	Have you ever consumed a drink that contains alcohol (such as beer, wine, etc.)?	453 (27.2%)
5.	Have you consumed alcohol in the last 30 days?	261(15.6%)
	**Physical activity**	
6.	Does your work involve vigorous- intensity activity, that causes large increases in breathing or heart rate, [like heavy lifting, digging or chopping wood] for at least 10 minutes continuously? (n = 1668)	108 (6.5%)
7.	How much time do you spend doing vigorous- intensity activities at work on a typical day? (n = 108)	
	≤ 60 minutes	84 (77%)
	>60 minutes	24 (23%)
8.	Do you do any moderate-intensity sports, fitness or recreational (leisure) activities that cause a small increase in breathing or heart rate?	168 (10.1%)
9.	How much time do you spend doing moderate intensity sports, fitness or recreational (leisure) activities on a typical day?	
	≤ 60 minutes	84 (25%)
	>60 minutes	126 (75%)
10.	Do you walk or use a bicycle (pedal cycle) for at least 10 minutes continuously to get to and from places?	63 (3.8%)
11.	How much time would you spend walking or bicycling for travel on a typical day? (n = 63)	
	≤ 60 minutes	42 (66.7%)
	>60 minutes	21 (33.3%)

### Child related characteristics

The data from the study on children reveals several interesting trends. More than half of the children included in the study were female, indicating a slight gender imbalance in the sample. The majority of these female children fell within the age group of 13–23 months, suggesting a concentration of participants in this particular age range. Additionally, a significant proportion of children were aged between 36–47 months, highlighting a diverse age distribution within the sample.

In terms of breastfeeding status, a majority of the children were exclusively breastfed, reflecting a positive trend in infant feeding practices. This indicates a high level of adherence to recommended breastfeeding guidelines among the participants.

When examining the minimum dietary diversity score of the children, it is notable that only about one fourth of them had achieved the minimum dietary diversity score by consuming five or more food groups out of seven. This finding suggests that there may be room for improvement in terms of ensuring a varied and balanced diet for these children.

In terms of birth order, the data shows that the majority of children were the second child in their family, indicating a common birth order pattern within the sample. On the other hand, a smaller proportion of children were identified as firstborn in their family, suggesting a lower representation of this birth order category. (See Table 4).

**Table 4:** Characteristics of children for double burden of malnutrition and its associated factors among mother-child pair at household level in Dire Dawa city, Eastern Ethiopia, 2022 (n 1,668).

S. No	Variable	Response category	Frequency (%)
1.	**Child’ sex**	Male	764(45.8)
		Female	904(54.2)
2.	**Child’ age in month**	6–12	208(12.5)
		13–23	619(37.1)
		24–35	234(14.0)
		36–47	199(11.9)
		48–59	408(24.5)
3.	**Exclusive breast feeding**	yes	1026(61.5)
		no	642(38.5)
4.	**Child minimum dietary diversity (CDDS)**	poor	1252(75.1)
		good	416(24.9)
5.	**Childs’ birth order**	First	285(17.1)
		second	729(43.7)
		third	351(21.0)
		Fourth & above	303(18.2)

### Prevalence of double burden of malnutrition among mother-child pair

The study revealed concerning rates of child malnutrition, with 20.2% of children experiencing wasting, 21.0% stunting, and 17.7% underweight. These figures underscore the significant burden of malnutrition among the child population studied. Additionally, maternal overweight/obesity was found to be prevalent in 20.1% of cases, highlighting a potential dual burden of malnutrition within the household.

Furthermore, the coexistence of malnutrition in children and mothers was also observed, with rates of coexisting malnutrition such as (OM/SC), (OM/WC), and (OM/UC) estimated at 8.5%, 7.0%, and 7.9%, respectively. The overall double burden of malnutrition among mother-child pairs was found to be 12.3% [95% CI: 10.7, 13.7]. This suggests a complex interplay of nutritional challenges within families, potentially indicating shared risk factors or environmental influences contributing to these patterns. The findings point to the need for targeted interventions addressing both child and maternal nutrition to improve health outcomes and reduce the prevalence of malnutrition in the population. (See Table 5).

**Table 5:** Prevalence of double burden of malnutrition and its associated factors among mother-child pair at household level in Dire Dawa city, Eastern Ethiopia, 2022 (n 1,668).

S. No	Indicator	Weighted percent	95% CI for weighted percent	
			LL	UL
1.	**Underweighted**	295 (17.7%)	15.9	19.5
2.	**Stunted**	351 (21.0%)	19.2%	22.9%
3.	**Wasted**	337 (20.2%)	18.3%	22.2%
4.	**Maternal Overweight/Obesity**	335 (20.1%)	18.2%	22.1%
**Co-existence of malnutrition**				
5.	**OM/SC**	8.5%	7.1%	9.8%
6.	**OM/UC**	7.9%	6.7%	9.2%
7.	**OM/WC**	7.0%	5.8%	8.2%
8.	**DBM (OM with SC/WC/UC)**	12.3%	10.7%	13.7%

### Factors associated with double burden of malnutrition among maternal-child pair

A total of fourteen explanatory variables, including marital status, mother’s education, mother’s occupation, number of under-five children, birth order, MDDS, HDDS, child sex, child age, exclusive breastfeeding, CDDS, mother’s age, household food insecurity, and maternal height stature, exhibited a p-value of less than 0.25 in bivariable analysis, rendering them eligible for inclusion in the multivariable analysis. However, the results of the multivariable analysis revealed that marital status (divorced), birth order, number of under-five children, MDDS, CDDS, household food insecurity, and maternal stature were significantly correlated with the double burden of malnutrition in mother-child pairs.

The likelihood of getting mother-child pair double burden of malnutrition were 1.80 times higher among divorced mother compared with married mothers [AOR = 1.80; 95% CI: 1.15, 2.82]. There was a significant positive association between being fourth or above child in birth order and developing mother-child pair double burden of malnutrition [AOD = 1.88; 95% CI:1.08, 3.26]. Those who had five or more under-five children in the household had 1.58 times odds of getting mother-child pair double burden of malnutrition [AOR = 1.58; 95% CI: 1.04, 2.39]. Those who had inadequate MDDS and CDDS respectively, had 2.76—and 8.66—times odds of getting mother-child pair double burden of malnutrition ([AOR = 2.76; 95% CI: 1.71, 4.45] & [AOR = 8.66; 95% CI: 4.85, 15.44]). The study participants those who were household food insecure had 3.68 times odds of having mother-child pair double burden of malnutrition [AOR = 3.68; 95% CI: 2.36, 5.75]. The odds of getting mother-child pair double burden of malnutrition were 2.39 times higher among short stature mothers compared with normal mothers [AOR = 2.39; 95% CI: 1.65,3.45]. (Table 6).

**Table 6:** Multivariable analysis of double burden of malnutrition and its associated factors among mother-child pair at household level in Dire Dawa city, Eastern Ethiopia, 2022 (n 1,668).

Variables	Response category	Frequency (%)	MCDBM		95%CL	
			Yes	No	COR	AOR
**Marital status**	Married	1219(73.1)	126	1,093	1.00	1.00
	Unmarried	88(5.3)	14	74	1.64[.90, 2.99]	1.05[.51, 2.16]
	Divorced	253(15.2)	58	195	2.58[1.82,3.64]	1.80[1.15, 2.82]
	Windowed	108(6.5)	7	101	.6[.27,1.32]	.47[.19, 1.18]
**Mothers’ educational Status**	Cannot read & write	132(7.9)	10	122	.36[.17, .73]	.46[.20, 1.05]
	Can read & write	342(20.5)	48	294	.7[.47, 1.09]	1.57[.92, 2.68]
	Primary (grade 1-8^th^)	474(28.4)	40	434	0.4[.26, .62]	1.12[.65, 1.93]
	Secondary (grade9-12^th^)	411(24.6)	50	361	.62[.40, .92]	1.09[.64, 1.85]
	College & above	309(18.5)	57	252	1.00	1.00
**Mothers’ occupation**	No job	144(8.6)	21	123	1.00	1.00
	Private employee	330(19.8)	29	301	.56[.30, 1.0]	.64[.30, 1.35]
	Government employee	135(8.1)	8	127	.36[.15, .86]	.42[.15, .1.13]
	Farmer	411(24.6)	41	370	.64[.36, 1.14]	.45[.22, .91]
	Merchant	510(30.6)	63	447	.82[.48, 1.40]	.65[.34, 1.24]
	Daily labor	138(8.3)	43	95	2.64[1.47,4.76]	1.17[.56, 2.42]
**Number of under-five children**	1–2	899(53.9)	103	796	1.00	1.00
	3–4	448(26.9)	30	418	.55[.36, .84]	.64[.40, 1.02]
	≥5	321(19.2)	72	249	2.23[1.60,3.11]	1.58[1.04,2.39]
**Childs’ birth order**	First	285(17.1)	34	251	1.00	1.00
	second	729(43.7)	73	656	.82[.53, 1.26]	.79[.47, 1.32]
	third	351(21.0)	40	311	.94[.58, 1.54]	.76[.43, 1.35]
	Fourth & above	303(18.2)	58	245	1.74[1.10,2.76]	1.88[1.08,3.26]
**MDDS**	good	555(33.3)	105	450		1.00
	poor	1113(66.7)	100	1,013	.42[.31, .56]	2.76[1.71,4.45]
**HDDS**	good	539(32.3)	117	422	1.00	1.00
	poor	1129(67.7)	88	1,041	.30[.22, .411]	1.38[.87, 2.21]
**Child’ sex**	Male	764(45.8)	71	693	1.00	1.00
	Female	904(54.2)	134	770	1.69[1.25,2.30]	
**Child’ age in month**	6–12	208(12.5)	56	152	1.00	1.00
	13–23	619(37.1)	43	576	.20[.13, .31]	
	24–35	234(14.0)	24	210	.31[.18,.52]	
	36–47	199(11.9)	43	156	.74[.47,1.18]	
	48–59	408(24.5)	39	369	.28[.18, .45]	
**Exclusive breast feeding**	yes	1026(61.5)	116	910	1.00	1.00
	no	642(38.5)	89	553	1.26[.93,1.69]	
**CDDS**	Good	416(24.9)	141	275	1.00	1.00
	Poor	1252(75.1)	64	1,188	.10[.06, .89]	8.66[4.85,15.4]
**Mother’s age in year**	15–24	180(10.8)	13	167	1.00	1.00
	25–29	529(31.7)	68	461	1.89[1.02,3.51]	
	30–34	792(47.5)	106	686	1.98[1.08,3.61]	
	≥35	167(10)	149	149	1.55[.73,3.27]	
**HFS**	Good	1126(67.5)	54	1,072	1.00	1.00
	poor	542(32.5)	151	391	7.66[5.50,10.8]	3.68[2.36,5.75]
**Maternal height stature**	Normal	960(57.6)	77	883	1.00	1.00
	short	708(42.4)	128	580	2.53[1.87,3.42]	2.39[1.65,3.45]

## Discussion

The double burden of malnutrition among mother-child pairs in Eastern Ethiopia was found to be 12.3% [95% CI: 10.7, 13.7]. The finding is comparable with findings from Egypt (10.9%) [1]. However, the finding is higher than rates reported in previous studies. National surveys in Brazil, China, and Russia showed prevalence rates of 11.0%, 8.0%, and 8.0% respectively [39]. In Latin American countries, 11.0% in Brazil, 9.3% in Bolivia, 3.2% in Colombia, and 6.7% in Peru [40]. Mexico had a rate of 8.4% (33), Nepal 6.60%, and South/Southeast Asian countries 10.0% [41]. India had a rate of 6% [42], in Ethiopia 5.1% [43]. One potential explanation for the high prevalence observed in the study area could be attributed to the fact that the research was carried out at the community level, focusing on populations in remote areas. The survey included a sample size of over 1500 participants, which may have contributed to the higher prevalence rates identified. Additionally, the high prevalence of stunting in Ethiopia [44] could also be a contributing factor. Research suggests that countries with elevated levels of under-nutrition are more susceptible to experiencing higher rates of obesity [45, 46], which could further exacerbate the prevalence of double burden of malnutrition among mother-child pairs in the study area.

On the other hand, Indonesia reported a rate of 30.6% [47], Peninsular Malaysia 29.6% [48], and the Gaza Strip, Palestine 15.7% [49], which were higher than the current finding. One potential explanation for this variation could be attributed to differences in socio-economic status, level of urbanization, and the stage of nutrition transition among the countries. Another possible factor contributing to the discrepancies in the prevalence of DBM across countries could be the varying study periods.

Recent studies have shed light on a concerning trend regarding the double burden of malnutrition within mother-child pairs, particularly among divorced women compared to their married counterparts. The research indicates that divorced mothers are 1.80 times more likely to face this burden than married mothers, highlighting a significant disparity in nutritional outcomes. This finding is consistent with research conducted in various regions, in India [50], Indonesia [51], Nepal [52] and Addis Ababa & Kersa district [53]. The higher likelihood of divorced women experiencing the double burden of malnutrition can be attributed to several factors. Married women generally have better access to food resources, which enables them to adopt healthier child feeding practices and reduce the risk of malnutrition in their children. In contrast, divorced women may encounter challenges in providing adequate food for their children, potentially due to factors like loneliness or other socioeconomic constraints. These difficulties can contribute to a higher prevalence of malnutrition and other health issues within households headed by divorced women.

According to the current finding, children born fourth or later were found to be 1.88 times more likely to experience the double burden of malnutrition within mother-child pairs at the household level, compared to children born first. This finding is consistent with research conducted in Addis Ababa and Kersa district [43], Bangladesh [54], and across 18 countries in Sub-Saharan Africa [55]. The increased vulnerability of later-born children to sub-optimal nutrition and health outcomes is a contributing factor to this disparity [35]. Additionally, as the number of births in a family increases, the available food and resources per household member decrease, potentially leading to higher-order births being more susceptible to malnutrition and other health issues [54].

This study also found that, the odds of having DBM among mother child pair for mothers with poor child dietary diversity was 2.76 times higher than mothers with good child dietary diversity. This result is concord with other studies done in India [56] and Addis Ababa & Kersa district [43]. This could be attributed to the impact of early nutritional exposure on adult health. Initially, a diet low in energy density due to fat restrictions may reduce serum leptin levels, leading to metabolic adaptations that could contribute to obesity and leptin resistance later in life. These effects may be influenced, in part, by epigenetic mechanisms [57]. The importance of ensuring adequate nutrition during the critical first 1000 days of life, including gestation and the first two years after birth, as well as promoting good preconception nutrition, is emphasized as a way to break the cycle of malnutrition across generations and address the coexistence of multiple forms of malnutrition in later life. The narrative underscores that early childhood presents a unique opportunity for parents and caregivers to provide essential nutrition, shape food preferences, and instill dietary habits that can have long-lasting effects on an individual’s health throughout their lifetime [58].

In addition, this study showed that, the odd of having DBM among mother child pair for children who had poor CDDS was 8.66 times higher as compared to those children with good CDD. This might be due to the fact that, the impact of early-life nutrition plays a crucial role in determining the risk of childhood and adult obesity, as well as setting the stage for the coexistence of multiple forms of malnutrition. Early childhood presents a special opportunity for parents and caregivers to provide essential nutrition and influence food preferences and dietary habits that can have long-lasting effects [59]. Improving the diversity of complementary foods can help reduce malnutrition, as dietary diversity is a reliable indicator of dietary quality and micronutrient density in children [60, 61].

The current study also found that, the study participants those who were household food insecure was 3.68 times high likely to have mother-child pair double burden of malnutrition. This could be attributed to the interplay between malnutrition and obesity, where both conditions are influenced by unhealthy dietary habits, particularly in the context of food insecurity [62, 63]. Research finding [64] highlights a complex interplay between malnutrition and obesity, indicating that individuals who experience early-life undernutrition may be at a higher risk of developing obesity in adulthood. This connection is particularly worrisome for populations already struggling with chronic hunger, as they not only suffer the effects of inadequate nutrition during infancy but also face a heightened susceptibility to obesity and related health issues, such as cardiovascular diseases, as they grow older. This dual burden of malnutrition experienced by both mothers and their children underscores the urgent need for comprehensive interventions that address nutritional deficiencies at different life stages to break the cycle of malnutrition and promote long-term health and well-being for vulnerable populations.

The study confirms that mothers with short stature are 2.39 times more likely to have mother-child pairs Double Burden Malnutrition (MCDBM) compared to women of normal height. This association could be attributed to the significant role maternal height plays in reflecting the intergenerational connection between maternal and child nutrition and health [65]. Short maternal stature is linked to an increased risk of stunting and wasting in children under the age of five, highlighting the importance of addressing this intergenerational connection to enhance maternal and child nutrition outcomes and reduce stunting in young children [66]. During the critical growth period in the first two years of life, nutritional deficiencies can lead to various outcomes depending on the nature of the deficiency. Acute malnutrition can result in weight loss and emaciation in children, while chronic undernutrition can impede linear growth, potentially leading to stunting. Chronic undernutrition limits children’s growth potential and may result in adults with reduced stature, increasing their susceptibility to multiple forms of malnutrition [67].

### Limitation

This study has limitations, one of which is its cross-sectional design, akin to the egg-chicken dilemma. Additionally, not all variables were directly measured; instead, many were self-reported, which can lead to potential issues such as exaggeration, embarrassment in disclosing private information, and biases influencing the results. Furthermore, recall bias was a concern in this study as some variables relied on the mothers’ memory, potentially affecting the accuracy of the data.

## Conclusion and recommendation

The research findings suggest that there is a concerning prevalence of the double burden of malnutrition affecting both mothers and children within households in the study area. This phenomenon poses a significant public health challenge, as it indicates that both mothers and children are experiencing malnutrition concurrently. Factors such as marital status, higher childbirth order, a greater number of under-five children in the household, low maternal and child dietary diversity scores, household food insecurity, and short maternal stature were identified as significant contributors to this issue. These findings underscore the complex and multifaceted nature of malnutrition among mother-child pairs, highlighting the need for targeted interventions to address this critical public health concern and improve the nutritional status of both mothers and children in the community. Early life nutrition is critical in shaping the risk of childhood undernutrition and adult obesity, leading to the mother-child pair double burden of malnutrition. Prevention efforts should start early, focusing on the first 1000 days of a child’s life. Breaking the intergenerational cycle of malnutrition is essential. Policy makers must prioritize improving child and maternal nutrition, emphasizing early life and preconception nutrition to address the mother-child pair double burden of malnutrition.

## Abbreviations

AOR: Adjusted Odds Ratio
BMI: Body Mass Index
CDDS: Child Dietary Diversity Score
CI: Confidence Interval
DBM: Double Burden of Malnutrition
DDS: Dietary Diversity Score
FAO: Food and Agricultural Organization
HDDS: House-hold Dietary Diversity Score
HFIAS: Household Food Insecurity Access Scale
MCDBM: Mother-child pair Double Burden of Malnutrition
MDDS: Maternal Dietary Diversity Score
MIYCN: Maternal Infant and Young Child Nutrition
NCDs: Non-Communicable Diseases
OR: Odds Ratio
OWM: Over Weight Mother
OWOBM: Over Weight or Obese Mother
UWC: Under Weight Child
USWC: Under Weight or Stunted or Wasted Child
WHO: World Health Organization
